# Analysis of risk factors for venous thromboembolism in hospitalized patients with acute herpetic neuralgia and construction of a predictive nomogram model

**DOI:** 10.3389/fneur.2026.1877838

**Published:** 2026-10-20

**Authors:** Chunman Wang, Guiling Wang, Bing Liu, Qian Gao, Chuansui Du, Lin Li

**Affiliations:** Pain Department, Hengshui People's Hospital, Hengshui, Hebei, China

**Keywords:** AHN, LASSO, prediction model, risk factors, VTE

## Abstract

**Objectives:**

To explore the risk factors for venous thromboembolism (VTE) in hospitalized patients with acute herpetic neuralgia (AHN) and to construct a VTE prediction model based on clinical indicators to help clinicians better identify high-risk patients and develop prevention and treatment strategies.

**Materials and methods:**

This study retrospectively included 327 patients diagnosed with AHN who were hospitalized in the Pain Department of Hengshui People's Hospital between December 2020 and December 2023, of whom 26 developed VTE during hospitalization. The basic clinical data of the patients were collected, and the independent risk factors for VTE were screened by LASSO regression and multifactorial Logistic regression analysis, and the VTE prediction model was constructed. Model performance was evaluated by ROC curves, calibration curves, and decision curves, and internal validation was performed using ten-fold cross-validation and bootstrap resampling.

**Results:**

The incidence of VTE in AHN patients was 7.95%. LASSO regression screened for 10 relevant variables, and final multifactor logistic regression analysis identified FIB (fibrinogen), FDP (fibrin degradation products), NRS (Numerical Rating Scale) score, and thrombotic risk score as independent predictors. Among them, FIB was a protective factor for VTE (OR = 0.477, *P* = 0.006), while FDP (OR = 1.48, *P* < 0.001), NRS score (OR = 1.584, *P* = 0.039), and thrombotic risk score (OR = 2.05, *P* < 0.001) were risk factors. The AUC of the prediction model was 0.853 (95% CI: 0.764–0.941), and the bootstrap optimism-corrected C-index was 0.834. At the optimal cutoff, the sensitivity and specificity were 0.731 and 0.877, respectively, with good calibration and clinical applicability.

**Conclusions:**

In this study, a VTE prediction model was constructed for patients with AHN. The model can accurately predict the risk of VTE in patients with AHN and provide a reference for clinical prevention and intervention; however, external validation in larger, multicenter cohorts is warranted before clinical implementation.

## Introduction

Acute herpetic neuralgia (AHN) refers to the pain caused by the varicella-zoster virus (VZV) infecting sensory nerve fibers and the skin, resulting in the appearance of herpes and pain in the corresponding dermatomes. The pain typically occurs within 1 month of the onset of the rash (1). AHN is a common clinical condition, characterized by the simultaneous presence of herpes on the skin and associated neuralgia (2).

Venous thromboembolism (VTE) encompasses both deep venous thrombosis (DVT) and pulmonary thromboembolism (PTE). The occurrence of VTE not only causes significant discomfort and increases the risk of death and disability for patients, but also prolongs hospital stays, raises medical costs, and imposes a heavy burden on both society and the patient's family. Therefore, the prevention of VTE in clinical practice is of critical importance. There is a close association between the occurrence of AHN and VTE (3). AHN patients often share these characteristics, such as a higher prevalence among the elderly, the presence of malignancy in some cases, reduced activity due to pain, and the need for minimally invasive treatments. These features closely overlap with the risk factors for VTE, highlighting the importance of VTE prevention and management in AHN patients. Studies by Bubak et al. (4) and Fiordelisi et al. (5) have demonstrated that patients with VZV infections have a significantly higher incidence of vascular risks, including VTE, compared to those with other diseases. Additionally, research by Mackman and colleagues (6) suggests that viral infections can promote thrombosis by inducing cells to express tissue factor and by enhancing the release of tissue factor-positive extracellular vesicles. The severe pain caused by AHN may also contribute to decreased patient mobility, leading to reduced blood flow in the lower extremities, blood stasis, and increased thrombus formation (7). Furthermore, the use of corticosteroids in the treatment of acute herpes zoster may result in water and sodium retention, further promoting blood stasis and increasing the risk of thrombosis (8, 9).

Currently, research on the complications of VTE in patients with AHN remains limited. Existing VTE risk assessment models for general medical inpatients (e.g., Padua, IMPROVE/IMPROVE-DD) were developed in broader hospitalized populations and have not been specifically evaluated in AHN patients, who may have additional disease-specific drivers such as virus-related prothrombotic pathways and pain-related immobility. Therefore, this study aims to develop a VTE prediction model specifically for AHN patients using routine clinical indicators. This model will assist clinicians in enhancing their awareness of VTE risk in AHN patients. Additionally, it will help in formulating better prevention and treatment strategies for high-risk patients, reducing the incidence of VTE, and improving overall patient outcomes.

## Materials and methods

### Statement regarding informed consent

This retrospective study was approved by the Ethics Review Committee of Hengshui People's Hospital (approval number: 2022-2-019-1) and was conducted in accordance with the principles of the Declaration of Helsinki. Because the study involved only a retrospective review of de-identified medical records and posed no more than minimal risk to participants, the requirement for individual informed consent was waived by the Ethics Review Committee, in accordance with institutional policy and national legislation. Patient privacy and data confidentiality were fully protected throughout the research process.

### Diagnostic criteria for AHN

According to the International Classification of Diseases, 10th Revision (ICD-10) diagnostic criteria for herpes zoster, patients must present with clinical manifestations of herpes zoster, including unilateral erythema, papules, or pustules on the skin, accompanied by significant neuralgia. Acute neuralgia is defined as pain that persists within the first month following the appearance of the herpes zoster rash, with a Numerical Rating Scale (NRS) score >4 (1).

### Diagnostic criteria for VTE

VTE events occurring during hospitalization were confirmed by objective imaging studies only: DVT by compression or duplex ultrasonography, and pulmonary embolism (PE) by computed tomography pulmonary angiography (CTPA) or, when CTPA was contraindicated, ventilation/perfusion (V/Q) scanning. All imaging results were interpreted by experienced radiologists or vascular surgeons. Although D-dimer levels and clinical assessment were recorded, consistent with current guidelines, neither elevated D-dimer nor clinical suspicion alone was used to establish the diagnosis of VTE.

### Inclusion criteria

(1) We included patients with AHN whose index visit occurred within 30 days after herpes zoster rash onset. (2) Age ≥ 20 years. (3) Complete clinical and laboratory data available from the index hospitalization.

### Exclusion criteria

(1) Patients with a history of VTE. (2) Patients currently on anticoagulant or antiplatelet medications. (3) Patients with severe hepatic or renal dysfunction (e.g., ALT/AST levels more than twice the upper normal limit, eGFR < 30 mL/min/1.73 m^2^). (4) Patients with known active malignancy or who have recently undergone chemotherapy/radiotherapy. (5) Pregnant or breastfeeding women. (6) Patients with other severe systemic diseases or unstable conditions (e.g., myocardial infarction, stroke, infection). (7) Patients unable to complete the follow-up requirements of this study.

### Data collection

A retrospective review was conducted from December 2020 to December 2023 on 327 patients diagnosed with AHN in the Pain Department of Hengshui People's Hospital, 26 of whom developed VTE. Clinical data collected from AHN patients, both those with and without VTE, included demographic characteristics [age, sex, and body mass index (BMI)], comorbidities [hypertension, diabetes, coronary heart disease, cerebral infarction, cancer, renal insufficiency, chronic obstructive pulmonary disease (COPD), electrolyte disturbance, rheumatism and immunity, and atrial fibrillation], laboratory indicators [platelet count, prothrombin time (PT), activated partial thromboplastin time (APTT), fibrinogen (FIB), plasma antithrombin III (ATIII), D-dimer, and fibrin degradation products (FDP)], pain score [Numerical Rating Scale (NRS)], and the thrombosis risk score. These data were collected at baseline (T0), defined as the date of initial diagnosis or hospital admission. All laboratory indicators were obtained from the first blood samples drawn after admission, before the diagnosis of VTE and before the initiation of any anticoagulant therapy. The thrombosis risk score is the VTE risk assessment routinely performed by nursing staff at hospital admission in our institution (an institutional adaptation of the Caprini risk assessment model), recorded as an integer score ranging from 0 to 8 in this cohort, with higher values indicating higher estimated VTE risk; individual Caprini items were not systematically documented, so the score was analyzed as an aggregate admission assessment rather than as a formally reconstructed Caprini score. In-hospital pharmacologic or mechanical VTE prophylaxis was not systematically recorded in this retrospective dataset. The outcome was in-hospital VTE, defined as objectively confirmed VTE events occurring during the index hospitalization (from admission to discharge or in-hospital death); no post-discharge follow-up was included.

## Statistical methods

All statistical analyses and visualizations were performed using R version 4.3.3. The “CBCgrps” package was used to generate the baseline characteristics table. Group differences for categorical variables were analyzed using the chi-square test or Fisher's exact test. Continuous variables following a normal distribution were expressed as mean (±standard deviation) and compared between groups using the *t*-test, while non-normally distributed data were expressed as median (interquartile range) and analyzed using the rank-sum test. Group comparison results were expressed using *P*-values. Missing data were screened for all candidate variables; the final analytic dataset contained no missing values for any candidate predictor, and a complete-case analysis was therefore performed. A correlation heatmap between variables was calculated and plotted using the “corrplot” package, and multicollinearity among predictors retained in the final model was assessed using variance inflation factors (VIF), with VIF < 5 considered acceptable. Variable selection was based on the Lasso regression model, with continuous variables standardized using the min-max scaling method before entering the Lasso model. Lasso regression was performed using the “glmnet” package (with nfolds set to 10) to select the optimal variables. Given the limited number of VTE events, the prediction model was developed on the full cohort (*n* = 327), and internal validation was performed using 10-fold cross-validation and bootstrap resampling (1,000 repetitions) to obtain optimism-corrected estimates of model performance, as recommended for prediction-model development in low-event settings. The “Rms” package was used to establish the logistic regression model, with variable selection based on the Lasso regression results; variables selected by LASSO were entered into a multivariable logistic regression model and further refined by stepwise selection in both forward and backward directions based on the Akaike information criterion (AIC). Non-significant variables were excluded from the model to optimize it. The “caret” package was used to draw the calibration curve for the prediction model, and the ROC curve was calculated and plotted using the “pROC” and “ggplot2” packages. Model discrimination was evaluated using the area under the receiver operating characteristic curve (AUC) and the concordance index (C-index). At the optimal cutoff determined by the Youden index, a confusion matrix was constructed, and sensitivity, specificity, positive predictive value (PPV), negative predictive value (NPV), and accuracy were calculated with 95% confidence intervals. Model calibration was assessed using calibration curves and the Hosmer–Lemeshow goodness-of-fit test, and clinical utility was evaluated by decision curve analysis (DCA). All analyses reported in this manuscript, including Tables 1, 2, and Figures 1–4, were generated from the same verified analysis dataset; after the correction of the descriptive table, the entire analysis pipeline was re-run, and the variables selected by LASSO regression and the four predictors retained in the final model were unchanged.

**Table 1:** Baseline characteristics of the study participants.

Variable	Total (*n* = 327)	Non-VTE (*n* = 301)	VTE (*n* = 26)	*p* value
Sex (male), *n* (%)	153 (46.8)	145 (48.2)	8 (30.8)	0.133
Age (years), median [Q1, Q3]	66 [57.5, 73]	65 [57, 72]	75 [69.2, 77.5]	< 0.001
BMI (kg/m^2^), median [Q1, Q3]	24.2 [22.5, 26.7]	24.2 [22.5, 26.7]	23.9 [21.9, 25.9]	0.540
Hypertension, *n* (%)	157 (48.0)	141 (46.8)	16 (61.5)	0.217
Diabetes, *n* (%)	78 (23.9)	72 (23.9)	6 (23.1)	1.000
Coronary heart disease, *n* (%)	64 (19.6)	58 (19.3)	6 (23.1)	0.832
Cerebral infarction, *n* (%)	39 (11.9)	35 (11.6)	4 (15.4)	0.531
Cancer^a^, *n* (%)	20 (6.1)	18 (6.0)	2 (7.7)	0.666
Renal insufficiency, *n* (%)	6 (1.8)	6 (2.0)	0 (0.0)	1.000
COPD, *n* (%)	39 (11.9)	35 (11.6)	4 (15.4)	0.531
Electrolyte disturbance, *n* (%)	17 (5.2)	15 (5.0)	2 (7.7)	0.635
Rheumatism and immunity, *n* (%)	4 (1.2)	4 (1.3)	0 (0.0)	1.000
Atrial fibrillation, *n* (%)	6 (1.8)	5 (1.7)	1 (3.8)	0.394
Elevated D-dimer^b^, *n* (%)	55 (16.8)	40 (13.3)	15 (57.7)	< 0.001
PT (s), median [Q1, Q3]	10.8 [10.3, 11.4]	10.8 [10.3, 11.4]	10.9 [10.2, 11.2]	0.824
APTT (s), median [Q1, Q3]	26.8 [25.0, 28.5]	26.9 [25.1, 28.5]	25.3 [23.6, 27.2]	0.012
FIB (g/L), median [Q1, Q3]	2.9 [2.5, 3.5]	2.9 [2.5, 3.5]	2.7 [2.4, 3.6]	0.505
FDP (mg/L), median [Q1, Q3]	2.0 [1.5, 2.9]	1.9 [1.5, 2.7]	4.3 [2.5, 9.1]	< 0.001
ATIII (%), median [Q1, Q3]	92.0 [84.0, 102.0]	92.0 [84.0, 102.0]	97.5 [85.2, 103.8]	0.309
Platelet count (× 10^9^/L), median [Q1, Q3]	217.0 [172.0, 266.5]	217.0 [172.0, 264.0]	231.0 [174.2, 271.0]	0.440
Pain score (NRS), median [Q1, Q3]	7 [6, 8]	7 [6, 8]	8 [7, 8]	0.002
Thrombosis risk score, median [Q1, Q3]	2 [1, 3]	2 [1, 3]	3 [3, 3]	< 0.001

^a^Cancer definition: history of malignancy without active disease or ongoing treatment (consistent with exclusion criteria).

^b^D-dimer cutoff: >0.5 mg/L FEU (institutional reference range).

Data presented as *n* (%) for categorical variables; median [Q1, Q3] for continuous variables. Categorical variables were compared using the chi-square test, or Fisher's exact test when expected cell counts were < 5 (cerebral infarction, cancer, renal insufficiency, COPD, electrolyte disturbance, rheumatism and immunity, atrial fibrillation, and elevated D-dimer); continuous variables were compared using the Mann–Whitney *U* test. Significant *p* values (< 0.05) are bolded.

**Table 2:** Multivariate binary logistic regression analysis of VTE in patients with AHN.

Variable	B	SE	Wald	OR(95%CI)	*P*
(Intercept)	−6.887	1.772	15.107	0.001(0–0.026)	< 0.001
FIB	−0.741	0.269	7.603	0.477(0.275–0.798)	0.006
FDP	0.392	0.077	26.077	1.48(1.282–1.737)	< 0.001
NRS	0.460	0.223	4.250	1.584(1.05–2.538)	0.039
Thrombosis risk score	0.718	0.177	16.505	2.05(1.455–2.947)	< 0.001

**Figure 1:**
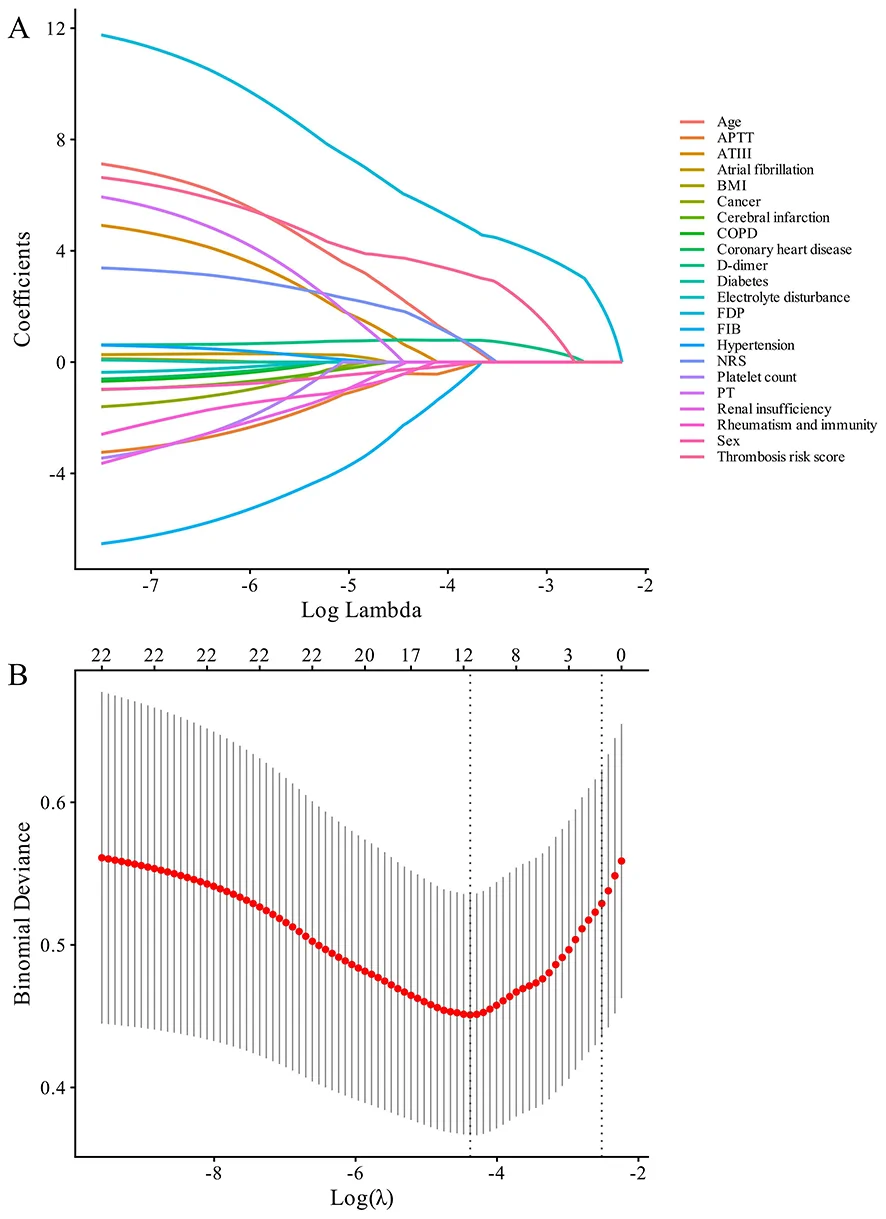
Variable selection via LASSO regression: **(A)** LASSO regression coefficient path plot. **(B)** cross-validation curve. The candidate variables shown correspond to those listed in Table 1.

## Results

### Basic information of patients

Based on the inclusion and exclusion criteria of this study, 327 patients with AHN were enrolled, of whom 26 developed VTE during hospitalization. The incidence of VTE in AHN patients was 7.95%. Table 1 presents the baseline characteristics of the study participants, comparing the VTE group with the non-VTE group across various included indicators. Compared with the non-VTE group, patients in the VTE group were significantly older and had significantly higher FDP levels, a higher proportion of elevated D-dimer, higher NRS scores, and higher thrombosis risk scores (all *P* < 0.05; Table 1).

### LASSO regression analysis for variable selection

LASSO regression was used to select variables from the clinical data. Figure 1A illustrates the LASSO regression coefficient path plot, where each path represents the trajectory of a variable's regression coefficient as the regularization parameter λ varies. Figure 1B displays the cross-validation curve used to determine the optimal λ value through 10-fold cross-validation. To achieve the best model fit, the λ value corresponding to the minimum mean squared error was selected (indicated by the vertical dashed line on the left side of Figure 1B). Through LASSO regression analysis, a total of 10 variables were selected: sex, age, rheumatism and immunity, D-dimer, APTT, FIB, FDP, ATIII, NRS, and thrombosis risk score.

## Collinearity assessment

The correlation heatmap of all candidate variables is shown in Figure 2. Correlations among the coagulation-related markers were weak to moderate: FDP correlated most strongly with elevated D-dimer (*r* = 0.582), consistent with their shared fibrinolytic biology, and correlated only weakly with FIB (*r* = 0.357). Correlations between the thrombosis risk score and other predictors were also weak (e.g., with NRS, *r* = 0.144). In the final prediction model, all variance inflation factors were below 1.6 (FIB: 1.512; FDP: 1.534; NRS: 1.018; thrombosis risk score: 1.071), indicating no meaningful multicollinearity. Notably, the strongest correlation in the heatmap was observed between age and the thrombosis risk score (*r* = 0.627, *P* < 0.001), which is expected because the score is an institutional adaptation of the Caprini model that incorporates age as a scoring component; significant but weaker correlations were also observed between age and several comorbidities (e.g., hypertension, *r* = 0.253). Thus, the age-related risk of VTE was largely captured by the thrombosis risk score during variable selection, which explains why age, despite differing significantly between the groups, was not retained as an independent predictor in the final model.

**Figure 2:**
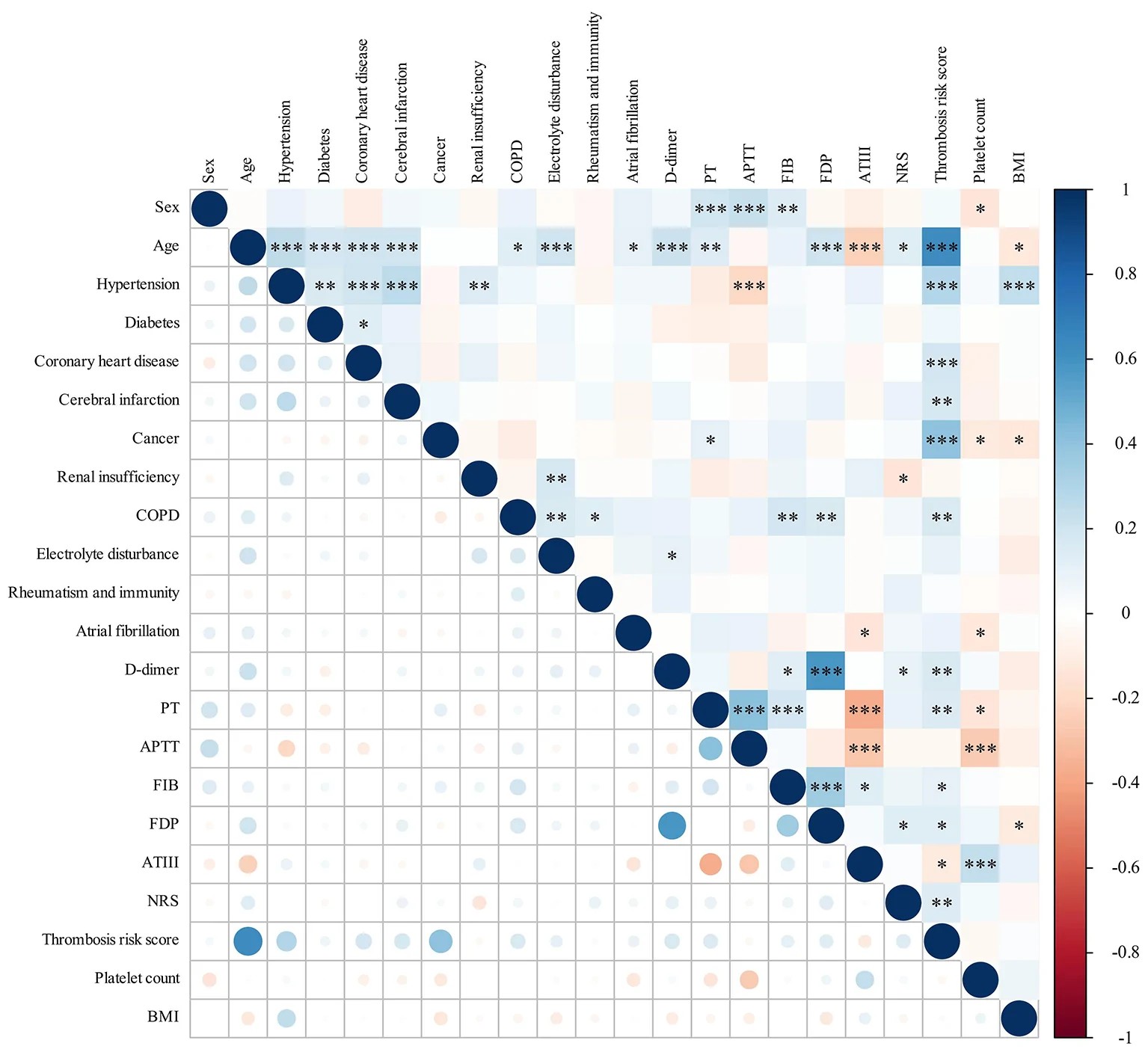
Correlation heatmap of candidate variables. Circle size and color indicate the strength and direction of Pearson correlation coefficients; asterisks denote statistical significance (*P < 0.05, **P < 0.01, ***P < 0.001).

## Multivariate logistic regression analysis

In univariate logistic regression analyses, elevated D-dimer (OR 8.898, 95% CI: 3.846–21.227, *P* < 0.001), age (OR 1.083 per year, 95% CI: 1.042–1.131, *P* < 0.001), APTT (OR 0.818 per second, 95% CI: 0.689–0.960, *P* = 0.018), FDP (OR 1.431 per mg/L, 95% CI: 1.246–1.665, *P* < 0.001), NRS score (OR 1.950 per point, 95% CI: 1.314–3.015, *P* = 0.002), and the thrombosis risk score (OR 1.908 per point, 95% CI: 1.412–2.652, *P* < 0.001) were significantly associated with VTE, whereas FIB was not (OR 1.025 per g/L, 95% CI: 0.631–1.516, *P* = 0.912). Based on the 10 variables selected through LASSO regression, a stepwise (both forward and backward, based on the Akaike information criterion) multivariate binary logistic regression was performed to identify independent risk factors and construct the final logistic regression model. The results are presented in Table 2: FIB (OR (95% CI): 0.477 (0.275–0.798), *P* = 0.006) was identified as an independent protective factor against VTE in patients with AHN. FDP (OR (95% CI): 1.48 (1.282–1.737), *P* < 0.001), NRS (OR (95% CI): 1.584 (1.05–2.538), *P* = 0.039), and thrombosis risk score (OR (95% CI): 2.05 (1.455–2.947), *P* < 0.001) were identified as independent risk factors for VTE in AHN patients.

## Development of a nomogram for predicting VTE in patients with AHN

Based on the results from the logistic regression analysis, we developed a clinical prediction model for VTE in patients with AHN, presented as a nomogram (Figure 3). The nomogram provides a visual tool for clinical practice, allowing for the calculation of the VTE risk probability in AHN patients. Using the nomogram, scores for each of the four predictive factors can be obtained from the scales for each variable. By summing these individual scores, the nomogram enables the estimation of the probability of VTE occurrence in AHN patients.

**Figure 3:**
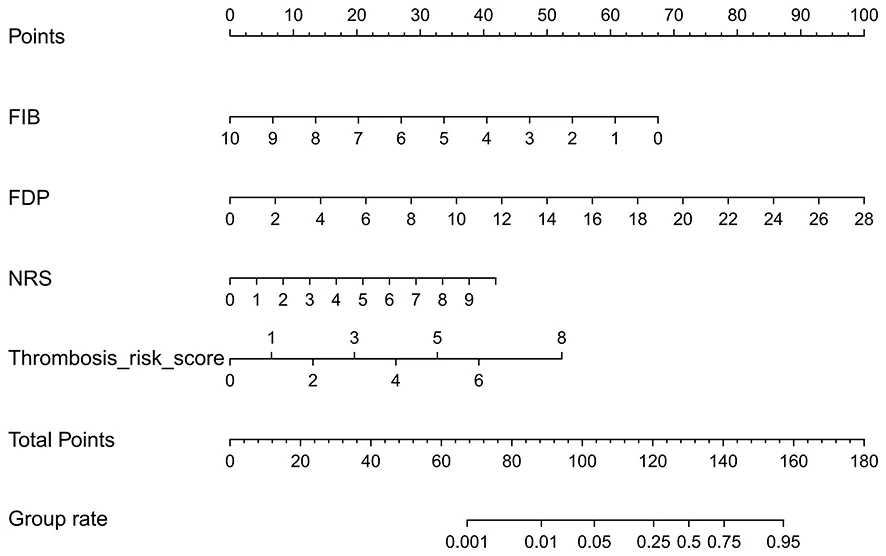
Nomogram for predicting VTE in patients with AHN based on logistic regression results.

## Evaluation of nomogram model performance

The apparent AUC of the prediction model in the full cohort was 0.853 (95% CI: 0.764–0.941) (Figure 4A). Internal validation using ten-fold cross-validation yielded a C-index of 0.849 (95% CI: 0.591–1.000), and bootstrap validation with 1,000 repetitions yielded an optimism-corrected C-index of 0.834, with the bootstrap distribution of the C-index showing a mean of 0.831 (95% CI: 0.680–0.950) (Figure 4B), indicating acceptable internal validity. At the optimal cutoff of 0.094 determined by the Youden index, the confusion matrix (Figure 4C) yielded 19 true positives, 7 false negatives, 264 true negatives, and 37 false positives, corresponding to a sensitivity of 0.731 (95% CI: 0.522–0.884), a specificity of 0.877 (95% CI: 0.835–0.912), a positive predictive value of 0.339 (95% CI: 0.218–0.478), a negative predictive value of 0.974 (95% CI: 0.948–0.990), and an accuracy of 0.865 (95% CI: 0.824–0.901). The calibration curve of the model (Figure 4D) indicates that the predicted probabilities closely align with the actual probabilities. The Hosmer–Lemeshow goodness-of-fit test yielded a χ^2^ value of 5.848 with a *P*-value of 0.664, suggesting that the difference between the predicted and actual values is not statistically significant (*P* > 0.05), indicating good model calibration. Figure 4E displays the decision curve analysis (DCA), which demonstrates that the model provides net benefits across most of the threshold range, indicating good clinical applicability.

**Figure 4:**
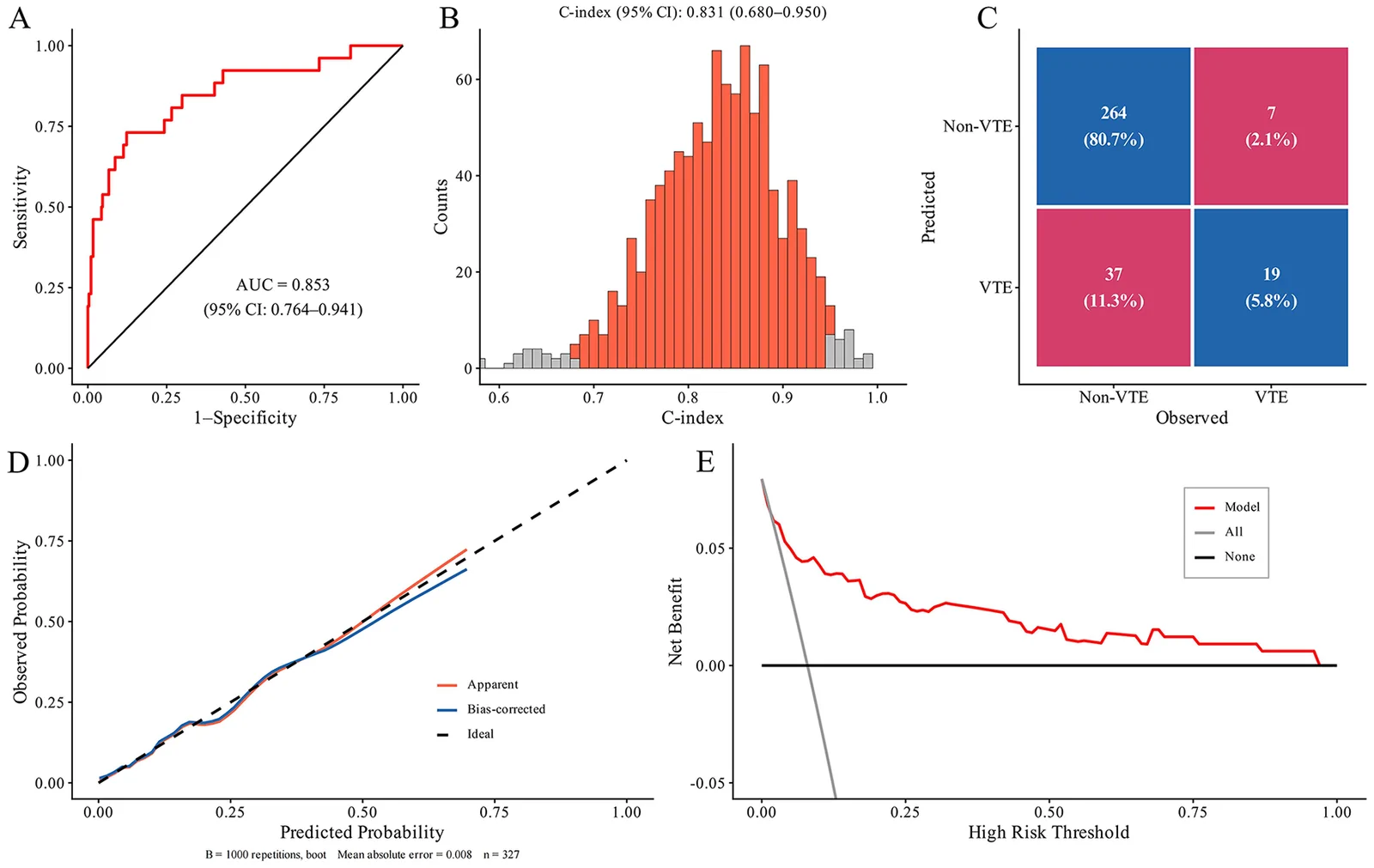
Evaluation of the prediction model performance: **(A)** ROC curve of the prediction model, with an AUC of 0.853 (95% CI: 0.764–0.941). **(B)** Distribution of the C-index across 1,000 bootstrap resamples (Mean 0.831, 95% CI: 0.680–0.950). **(C)** Confusion matrix of the prediction model at the optimal cutoff of 0.094. **(D)** Calibration curve of the prediction model, showing that the predicted probabilities closely align with the actual probabilities. **(E)** Decision curve analysis (DCA) of the prediction model, demonstrating good clinical applicability.

## Discussion

This study explores the relationship between AHN and VTE and develops a VTE prediction model for AHN patients using routine clinical indicators. The incidence of VTE among AHN patients was 7.95%. LASSO regression identified 10 relevant variables: sex, age, rheumatism and immunity, D-dimer, APTT, FIB, FDP, ATIII, NRS, and thrombosis risk score. Among these, FIB was identified as a protective factor for VTE (OR = 0.477, *P* = 0.006), while FDP (OR = 1.48, *P* < 0.001), NRS score (OR = 1.584, *P* = 0.039), and the thrombosis risk score (OR = 2.05, *P* < 0.001) were identified as risk factors. Using these four indicators, a prediction model was constructed and visualized with a nomogram. The model's AUC was 0.853, and the bootstrap optimism-corrected C-index was 0.834, and both the calibration curve and DCA indicated that the model provides a good fit for actual probabilities and has strong clinical applicability.

In this study, the incidence of VTE among AHN patients was 7.95%, which is consistent with previous research findings. D-dimer, a product of fibrin degradation in the blood, is commonly used to detect the presence of thrombus formation and fibrinolysis. It has high diagnostic sensitivity but lower specificity and is typically used as an adjunctive indicator for diagnosing VTE (10). In our study, patients in the VTE group were significantly older and had higher FDP levels, a higher proportion of elevated D-dimer, higher NRS scores, and higher thrombosis risk scores, aligning with previous studies that highlight hypercoagulability and reduced mobility as key triggers for VTE (11, 12). Additionally, some AHN patients received glucocorticoids during their treatment. Research indicates that glucocorticoids can exacerbate water and sodium retention, which further leads to venous blood flow stagnation and thrombus formation (8).

The results of the multivariate logistic regression analysis indicate that FIB, FDP, NRS, and the thrombosis risk score are independent predictors of VTE in AHN patients. Specifically, FIB acts as a protective factor against VTE, while FDP, NRS, and the thrombosis risk score are significant risk factors. However, this inverse association of FIB should be interpreted with caution: FIB was not significantly associated with VTE in the univariate analysis (OR 1.025 per g/L, 95% CI: 0.631–1.516, *P* = 0.912), and the inverse association emerged only after adjustment for FDP and other correlated coagulation markers. This pattern is more consistent with a suppression (negative confounding) effect among correlated coagulation indicators than with a truly protective biological action of fibrinogen. All coagulation indicators in this study were measured at admission, before the diagnosis of VTE and before the initiation of anticoagulant therapy, so reverse causation or treatment-related distortion is unlikely to explain this finding. This is consistent with the physiological mechanisms of VTE; an increase in FDP reflects active thrombus formation and breakdown, while a higher thrombosis risk score indicates an increased risk of thrombus development (13). Currently, there is a lack of research on the relationship between NRS scores and VTE. We hypothesize that severe pain may lead to reduced patient mobility, thereby increasing the risk of thrombus formation. Because bedrest time and mobility status were not systematically recorded in this retrospective dataset, this hypothesis could not be verified directly; however, the correlation between the NRS score and the thrombosis risk score was weak (*r* = 0.144), suggesting that the NRS score captures risk information beyond the admission risk assessment. The association between fibrinogen (FIB) and in-hospital VTE is biologically plausible because fibrinogen is an acute-phase reactant and a key substrate for fibrin formation; higher levels can enhance platelet aggregation and increase blood/plasma viscosity, thereby promoting a prothrombotic milieu (14). Consistent with this, the Leiden Thrombophilia Study reported a dose–response relationship in which markedly elevated fibrinogen levels were associated with substantially higher risk of first deep vein thrombosis (15). However, evidence is not uniform across populations, as a large prospective analysis (LITE) did not observe a significant association between total fibrinogen and incident VTE, suggesting that fibrinogen may act as a context-dependent marker reflecting concurrent inflammation/tissue injury rather than a standalone causal driver (16). It is also worth noting that although patients in the VTE group were significantly older, age was not retained as an independent predictor in the final model: age correlated strongly with the thrombosis risk score (*r* = 0.627, *P* < 0.001), which itself incorporates age as a scoring component, so the independent contribution of age was largely absorbed by the thrombosis risk score during variable selection (Figure 2).

The nomogram developed in this study demonstrated strong predictive capability, with an AUC of 0.853, a ten-fold cross-validated C-index of 0.849, and a bootstrap optimism-corrected C-index of 0.834, indicating substantial discriminative ability. The calibration curve shows that the predicted probabilities align closely with actual incidence rates. Additionally, the DCA suggests that the model has good applicability in clinical practice. These results suggest that the prediction model can assist clinicians in assessing the risk of VTE in AHN patients and guide the development of preventive strategies.

Finally, the positive predictive value of the model was modest (0.339 at the optimal cutoff): at a VTE incidence of 7.95%, most patients flagged as high-risk by the nomogram will not develop VTE (19 true positives vs. 37 false positives in this cohort). This is an expected consequence of the low base rate rather than a failure of discrimination, as reflected by the high negative predictive value (0.974). In clinical practice, the model should therefore be used to prioritize closer monitoring, repeated risk reassessment, and discussions about thromboprophylaxis in flagged patients, rather than as a stand-alone trigger for diagnostic work-up or treatment.

This study also has several limitations. First, the retrospective design may introduce bias, as the data depend on existing medical records. Second, the study was conducted at a single center, which may limit the generalizability of the results. Third, the number of VTE events was small (26 events), yielding an events-per-variable ratio below conventional thresholds and a substantial risk of overfitting; this instability is reflected in the wide confidence interval of the ten-fold cross-validated C-index (95% CI: 0.591–1.000). Although LASSO penalization, cross-validation, and bootstrap internal validation (optimism-corrected C-index = 0.834) were used to mitigate overfitting, the model should be regarded as hypothesis-generating and requires external validation before clinical use. Fourth, there was no universal VTE screening protocol in this cohort: imaging was performed based on clinical suspicion, so asymptomatic VTE events may have been missed and the reported incidence may be underestimated. Fifth, in-hospital pharmacologic or mechanical VTE prophylaxis was not systematically recorded, and residual confounding by prophylaxis use cannot be excluded. Sixth, bedrest time and mobility status were not recorded, precluding direct verification of the proposed pain–immobility–thrombosis pathway. Additionally, while this study identified several key risk factors for VTE in AHN patients, further research is needed to explore the underlying mechanisms of these associations, particularly the roles of pain and glucocorticoid use in VTE occurrence. Finally, individual Caprini risk assessment items were not systematically documented in this retrospective dataset; thus, we could not benchmark our model against a formally reconstructed Caprini score or other established risk assessment models. Future research should validate this prediction model in larger, multicenter, prospective studies, with standardized collection of prophylaxis, mobility, and structured risk-assessment data, to ensure its applicability to a broader population.

In summary, this study observed an increased risk of VTE in patients with AHN and identified several important clinical risk factors. The prediction model developed based on these factors may provide clinicians with an effective tool for assessing the VTE risk in AHN patients and for implementing timely preventive measures. Given the limited number of events and the single-center retrospective design, these findings should be regarded as hypothesis-generating and warrant external validation.

## Conclusion

We developed a VTE prediction model based on clinical indicators and visualized it using a nomogram. This model demonstrates high accuracy and good clinical applicability in predicting VTE occurrence in AHN patients. It may assist clinicians in early identification of high-risk individuals for VTE among AHN patients, thereby enabling better prevention and intervention strategies, reducing the incidence of VTE, and improving patient outcomes. However, given the small number of VTE events and the single-center retrospective design, external validation in larger, multicenter prospective cohorts is required before the model can be implemented in clinical practice.

## Data Availability

The raw data supporting the conclusions of this article will be made available by the authors, without undue reservation.
